# Estrogen Deficiency Leads to Further Bone Loss in the Mandible of CKD Mice

**DOI:** 10.1371/journal.pone.0148804

**Published:** 2016-02-17

**Authors:** Yuchen Guo, Ningyuan Sun, Xiaobo Duan, Xin Xu, Liwei Zheng, Dutmanee Seriwatanachai, Yongyue Wang, Quan Yuan

**Affiliations:** 1 State Key Laboratory of Oral Diseases, West China Hospital of Stomatology, Sichuan University, Chengdu, China; 2 Department of Oral Biology, Faculty of Dentistry, Mahidol University, Bangkok, Thailand; Université de Lyon—Université Jean Monnet, FRANCE

## Abstract

**Background:**

Chronic kidney disease (CKD) has been regarded as a grave public health problem. Estrogen is a critical factor for both renal protection and bone remodeling. Our previous study demonstrated that CKD impairs the healing of titanium implants. The aim of this study was to investigate the effects of estrogen deficiency on the mandibular bone in CKD mice.

**Methods:**

Forty eleven-week-old female C57BL mice were used in this study. Uremia and estrogen deficiency were induced by 5/6 nephrectomy and ovariectomy (OVX), respectively. After 8 weeks, the mice were sacrificed, and their mandibles were collected for micro-CT analysis and histological examination.

**Results:**

All the mice survived the experimental period. Serum measurements confirmed a significant increase in BUN in the CKD group that was further increased by OVX. OVX led to significant decreases in both the BV/TV and cortical thickness of the mandibular bone in CKD mice.

**Conclusion:**

In summary, our findings indicate that estrogen deficiency leads to further mandibular bone loss in CKD mice.

## Introduction

Chronic kidney disease (CKD), which is a grave health problem, affects millions of individuals worldwide [1–3]. Approximately 26.3 million Americans suffer from CKD [2], and the situation is similar in China. A recent survey found that 119.5 million Chinese have been diagnosed with CKD [1].

CKD is caused by reduced kidney function, which disturbs normal physiological mechanisms that regulate the blood levels of vitamin D, phosphate, calcium, parathyroid hormone (PTH), and fibroblast growth factor 23 (FGF23). Thus, CKD patients are predicted to develop impaired bone metabolism that leads to CKD mineral and bone disorder (CKD-MBD), which is characterized by cortical loss and trabeculation [4–6]. Such changes triggered by CKD were previously reported to alter mandibular bone remodeling [7, 8], eventually leading to jaw enlargement [9–11]. Moreover, periodontitis and periodontal bone loss were worse and more symptomatic in CKD patients compared with a healthy population [12–14].

Osteoporosis is marked by reduced bone strength, leading to an increased risk of bone fracture. Estrogen is a critical regulator of bone remodeling [15, 16]. By increasing osteoclast apoptosis, downregulating osteoclast precursors and stimulating osteoblast proliferation, estrogen maintains the balance between bone formation and resorption [17, 18]. Estrogen deficiency increases both bone formation and bone resorption but favors the latter, leading to decreased bone mineral density (BMD) and increased bone fracture risk [19–20]. Thus, estrogen deficiency is the greatest risk factor for postmenopausal osteoporosis [21]. Moreover, osteoporosis not only affects the skeleton but also impairs mandibular bone metabolism; this disease leads to decreases in the bone volume/tissue volume (BV/TV) ratio and trabecular thickness and an increase in trabecular separation [22, 23]. In dentistry, estrogen deficiency impairs the osseointegration of titanium implants, and this impairment was shown to be accompanied by lower bone-implant contact (BIC) ratio, BV/TV ratio, and biomechanical push-in resistance [24]. These effects resulted in poor bone healing and complications after titanium implant surgery [25, 26].

Meanwhile, estrogen plays an important protective role in the kidneys of CKD patients [27–30]; thus, estrogen loss accelerates the progression of CKD. Therefore, estrogen deficiency might lead to more severe CKD-MBD, with increased bone resorption and decreased trabecular BMD [31, 32]. It has been reported that estrogen administration can decrease the bone resorption rate and restore normal trabecular bone volume and trabecular connectivity in these CKD patients [33].

Though the effect of estrogen deficiency in the general population is mutually agreed upon [33, 34], its effect on the mandibular bone of CKD mice is still unclear. In addition, there has not been a focus on the effects of estrogen deficiency on the mandibular bone in CKD mice. Therefore, in the present study, we developed a CKD mouse model with and without estrogen deficiency to investigate mandibular bone structure.

## Materials and Methods

### Ethics Statement

This study was conducted in strict accordance with the recommendations in the Guide for the Care and Use of Laboratory Animals of the National Institutes of Health and the ARRIVE guidelines (http://www.nc3rs.org/ARRIVE). All the experiments were approved by the Ethics Committee of West China School of Stomatology, West China Hospital of Stomatology. All the surgeries were performed under anesthesia with an intraperitoneal injection of a combination of ketamine (100 mg/kg) and xylazine (10 mg/kg). In addition, buprenorphine (0.05 mg/kg) was given for perioperative analgesia to minimize pain and suffering.

### Animals

**Fig 1:**
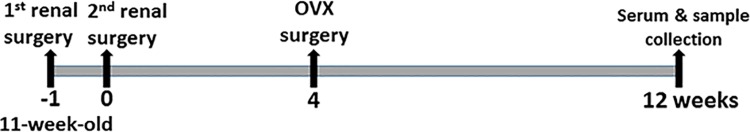
Illustration of the study workflow.

Eleven-week-old female C57BL mice were obtained from the Experimental Animal Center of Sichuan University and then randomly assigned to four groups: the Sham group (n = 10), the CKD group (n = 10), the OVX group (n = 10), and the CKD+OVX group (n = 10). The mice were housed in climate-controlled conditions (25°C, 55% humidity, and 12 hours of light alternating with 12 hours of darkness) and were fed a standard diet. The animal care and all the experiments were compliant with the guidelines of the Animal Research Committee of the West China School of Stomatology, Sichuan University, and were conducted in accordance with The Code of Ethics of the World Medical Association for Animal Experiments. The workflow for this study is shown in Fig 1.

### Surgical procedure to induce uremia

To induce uremia, a two-step 5/6 nephrectomy was performed on the mice in the CKD and CKD+OVX groups as previously described [35, 36]. Briefly, the first step involved electrocautery of the left kidney through a 2-cm-long lumbar incision on the dorsal aspect of the mouse after anesthesia. The entire cortex of the left kidney, except for a 2-mm area around the hilum, was cauterized (Acteon Servotome, Bordeaux, France). One week after the first surgery, total nephrectomy of the right kidney was performed after ligation of the renal hilum suture. Sham surgery included administering an anesthetic, creating a 2-cm-long lumbar incision, pushing out the kidney, guiding it back, and closing the abdominal wall. After the second renal surgery, all the mice were housed for four weeks to establish the uremia model.

### Ovariectomy

Four weeks after the second surgery for renal ablation, mice in the OVX and CKD+OVX groups underwent bilateral OVX as described previously [37]. Briefly, the ovaries were approached through a midline dorsal skin incision and exposed by cutting through the muscle layer right or left of the vertebral column. After ligating the uterine horn, both of the ovaries were surgically removed. Sham surgery included anesthesia, midline dorsal incision, exteriorizing the ovary, and wound closure.

### Serum chemistry

Twelve weeks after the second renal surgery, blood was collected from the cheek pouch of the mouse as described previously [38]. Serum biochemistry was assessed using the following commercially available kits: blood urea nitrogen (BUN) (Roche Diagnostics, Indianapolis, IN), calcium (0155–225, Stanbio Laboratory, Boerne, TX), phosphate (0830–125, Stanbio Laboratory, Boerne, TX), 1,25-dihydroxy vitamin D (1,25[OH]_2_D; IS-2400, Immunodiagnostic Systems Ltd., Fountain Hills, AZ), PTH (#60–2700, Immutopics, San Clemente, CA), and FGF23 (#60–6300, Immutopics, San Clemente, CA).

### Quantitative Analysis of Mandibles Using Micro-CT

After collecting blood from the mice, the animals were sacrificed with an overdose of isoflurane inhalation. Mandibles were collected for micro-CT and histological analyses. To prevent drying, all the mandibles were wrapped in parafilm during scanning. Mandibles were scanned on a micro-CT system (70 kV, 114 μA, 12 μm resolution; μ-CT 80 scanner, Scanco Medical, Bassersdorf, Switzerland). The volume of interest (VOI) was defined for each mandible from the reconstructed datasets. Between the mesial and distal roots, a buccal-lingual cross-slice of the first mandibular molar in the furcation zone was identified. Then, ten slices prior to and after the identified furcation slice were added to generate a VOI. Different thresholds were established to differentiate bone and other tissues. The lower and upper thresholds for bone were defined as 40 and 127 grayscale units. The BV/TV ratio and cortical thickness were quantitatively analyzed within the VOI.

### Histological Analysis

For the histological analysis, tissues were fixed in 10% buffered formalin for 1 week at 4°C and then transferred to 70% ethanol for storage. Mandibles were then dissected from the surrounding tissues and stored in a 17% EDTA solution for 4 weeks. Tissues were processed and embedded in paraffin. Five-micrometer frontal sections of the mandible were obtained. All the sections were stained with hematoxylin and eosin (H&E) as described previously [39]. Images of the furcation area between the mesial and distal roots of the mandibular first molar were obtained to observe the trabecular bone. Images of the middle and mesio-distal regions of the mesial root of the mandibular first molar were obtained to observe the cortical bone and PDL (periodontal ligament) space. Five slices prior to and after those images were analyzed. Nine points in each slice were chosen to measure the PDL space. The mean value was calculated and subjected to statistical analysis.

### Statistical analysis

All the data are presented as the mean ± SD. The Kolmogorov-Smirnov test was performed to confirm normality. One-way ANOVA was used to ascertain equality of variances and significant differences. Differences were considered significant at p<0.05.

## Results

### Serum chemistry

**Fig 2:**
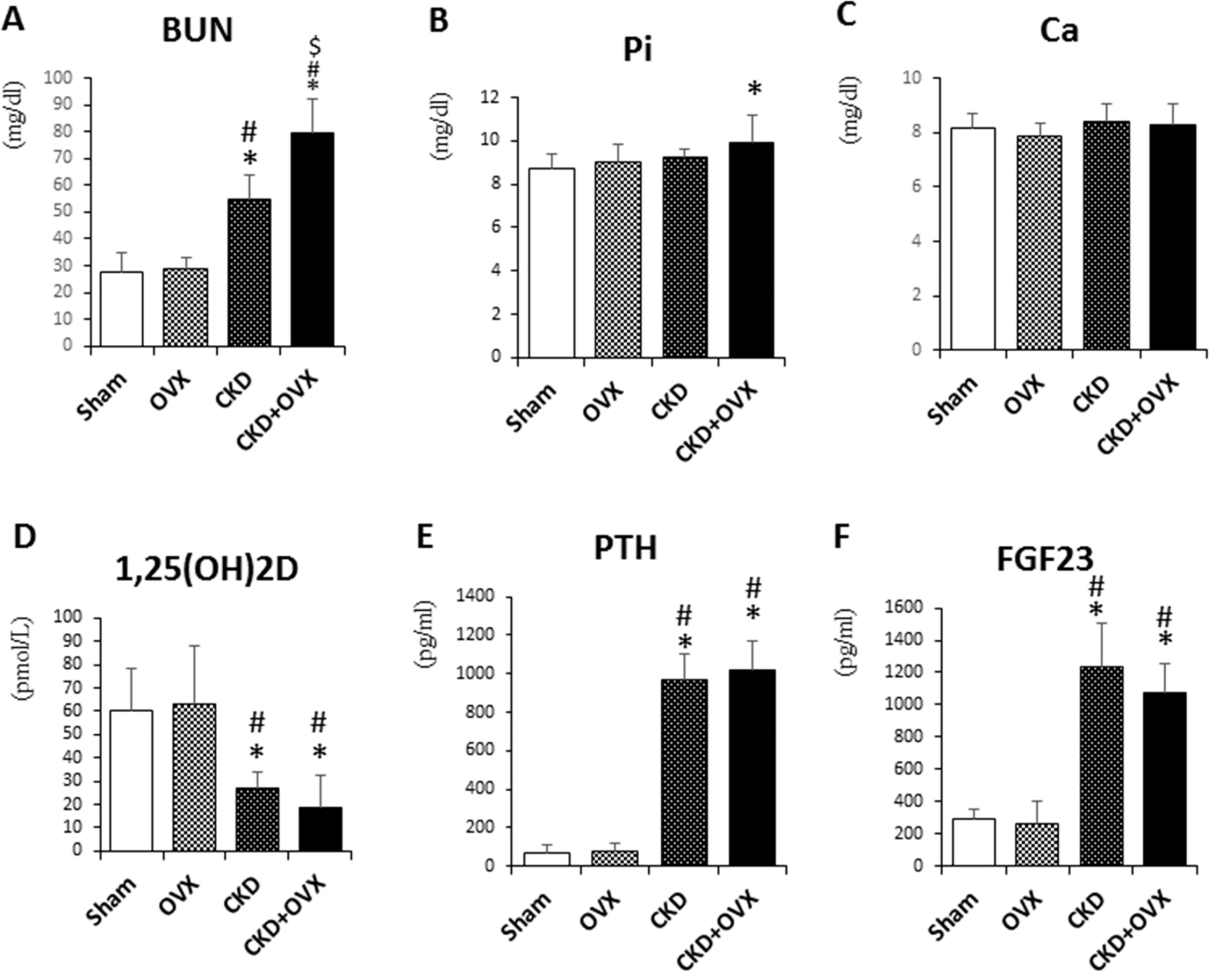
Serum biochemical measurements. (A) Serum BUN. (B) Serum phosphate. (C) Serum Calcium. (D) Serum 1,25(OH)2D. (E) Serum PTH. (F) Serum FGF23. *: p<0.05 vs Sham; #: p<0.05 vs OVX; $: p<0.05 vs CKD.

Serum BUN was significantly increased in the CKD group compared with the Sham group (p<0.05; Fig 2A), which indicated the creation of a uremic mouse model. Importantly, serum BUN was significantly higher in the CKD+OVX group than in the CKD group (p<0.05). Serum calcium levels were not different among the four groups (Fig 2C). Surprisingly, we observed a slight increase in serum phosphate in the CKD+OVX group compared with the Sham group (p<0.05; Fig 2B), but the CKD and OVX groups did not show any change in phosphate levels. As expected, serum 1,25(OH)_2_D levels were much lower in both CKD groups than in the Sham group, indicating that CKD disturbs normal renal function. Nevertheless, serum 1,25(OH)_2_D levels were not affected by OVX (Fig 2D). We also observed the development of hyperparathyroidism in both the CKD and CKD+OVX groups, which was thought to be secondary hyperparathyroidism induced by CKD. Consequently, serum FGF23 levels followed a similar pattern as PTH levels, as shown in Fig 2F.

### Micro-CT analysis

**Fig 3:**
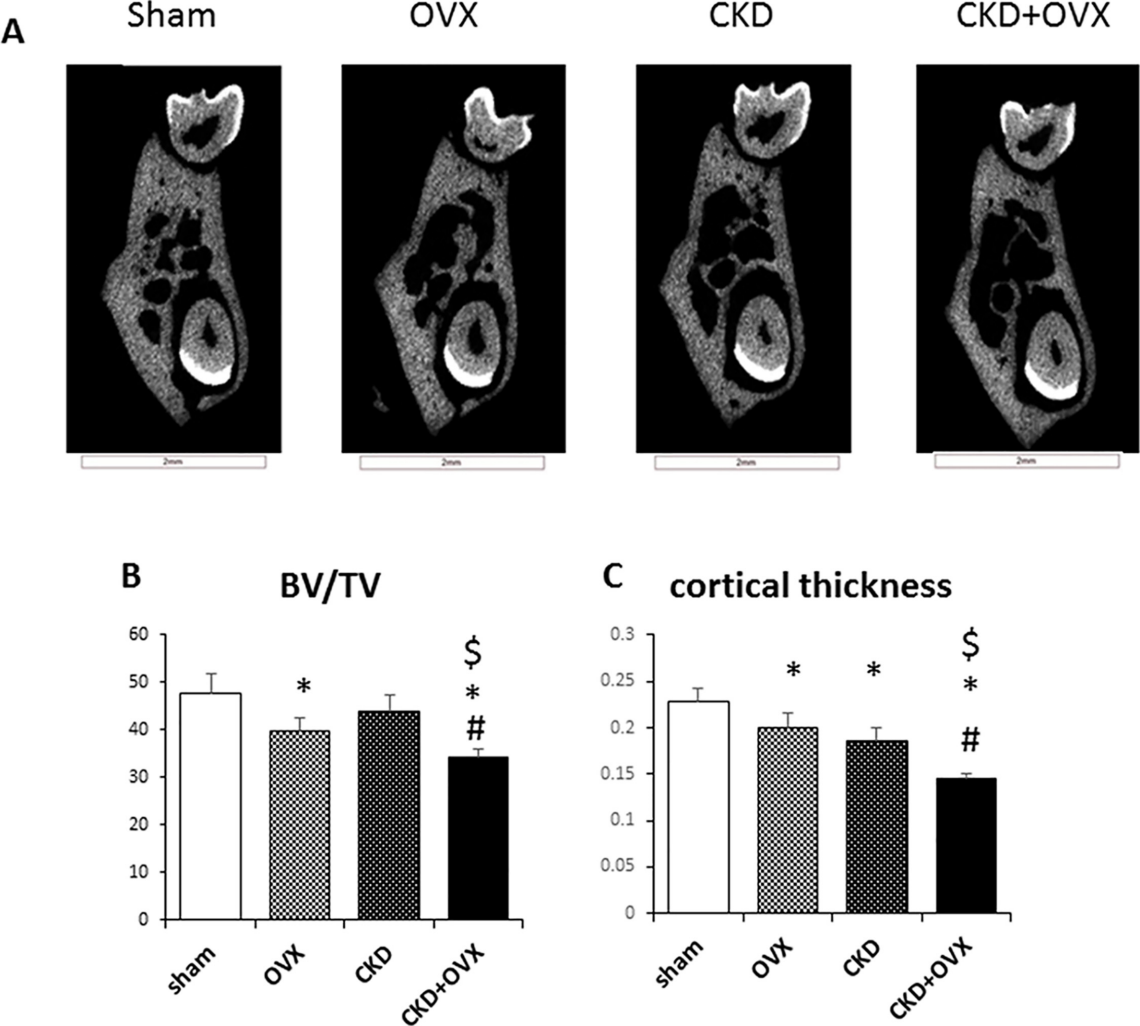
Tomographic cross-sectional slices of the furcation area and mandibular bone morphometry based on micro-CT analysis. (A) Representative mandibles from the Sham, OVX, CKD, and CKD+OVX groups. (B) Bone volume/tissue volume (BV/TV) ratio based on micro-CT analysis. (C) Cortical thickness based on micro-CT analysis. *: p<0.05 vs Sham; #: p<0.05 vs OVX; $: p<0.05 vs CKD.

Micro-CT was used to investigate microstructural changes in the mandibular bone of all the mice. One representative photo from each group is shown in Fig 3A. Mandibular bone loss was observed in the OVX group, as shown by the significant reductions in the BV/TV ratio and the cortical thickness (Fig 3B and 3C). We did not observe a significant decrease in the BV/TV ratio in the CKD group, but the cortical thickness was significantly reduced in this group compared with the Sham group. Notably, both parameters (i.e., BV/TV ratio and cortical thickness) were further reduced in the CKD+OVX group compared with the CKD group (p<0.05), and the CKD+OVX group exhibited the greatest bone loss among the four groups.

### Histological evaluation

**Fig 4:**
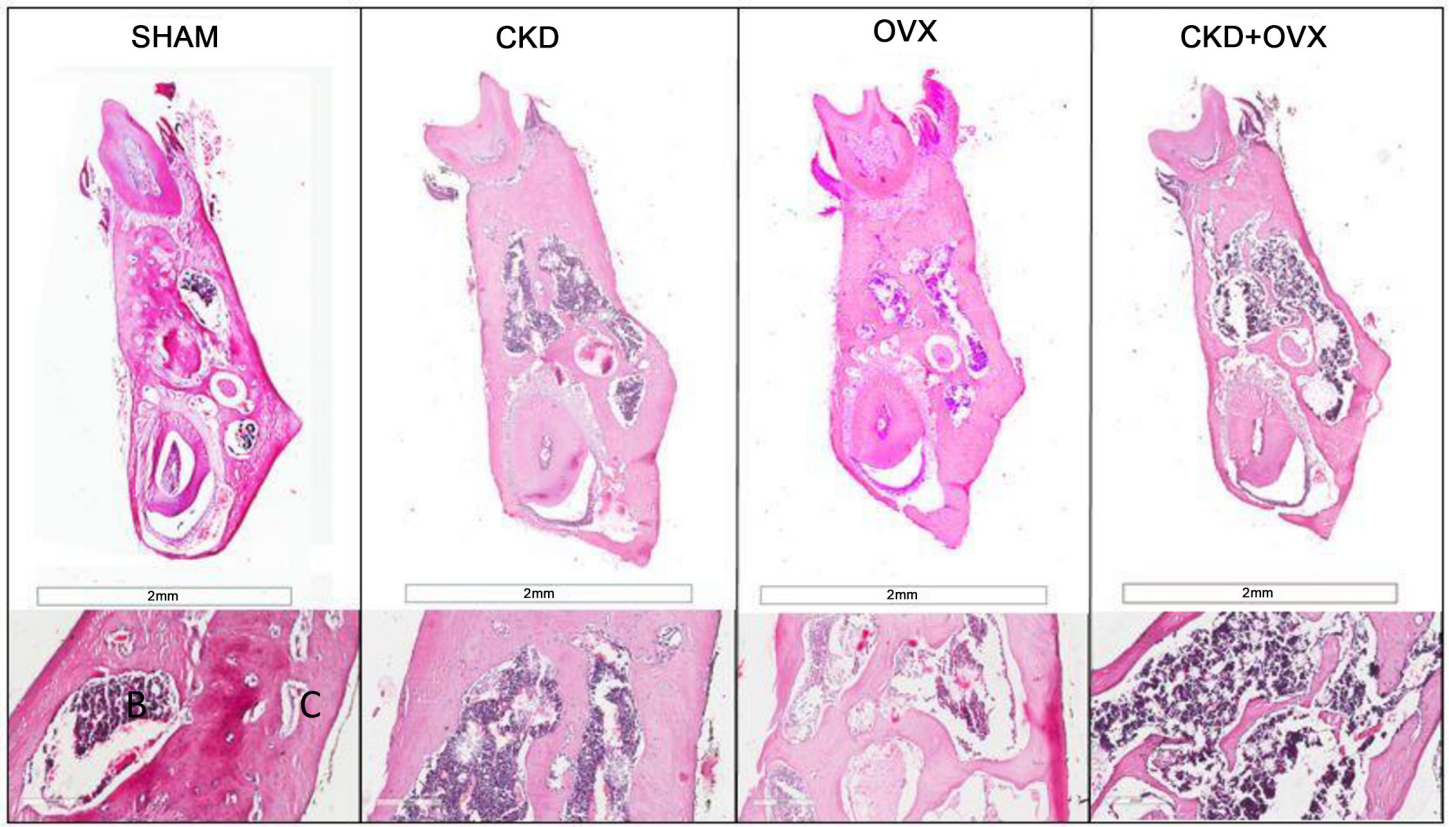
Histological staining of trabecular bone. (H&E staining) Buccal lingual cross sections of the furcation area between the mesial root and the distal root of the mandibular first molar. The second row consists of magnified images (400X) of trabecular bone. B: Bone marrow space; C: Cancellous bone.

**Fig 5:**
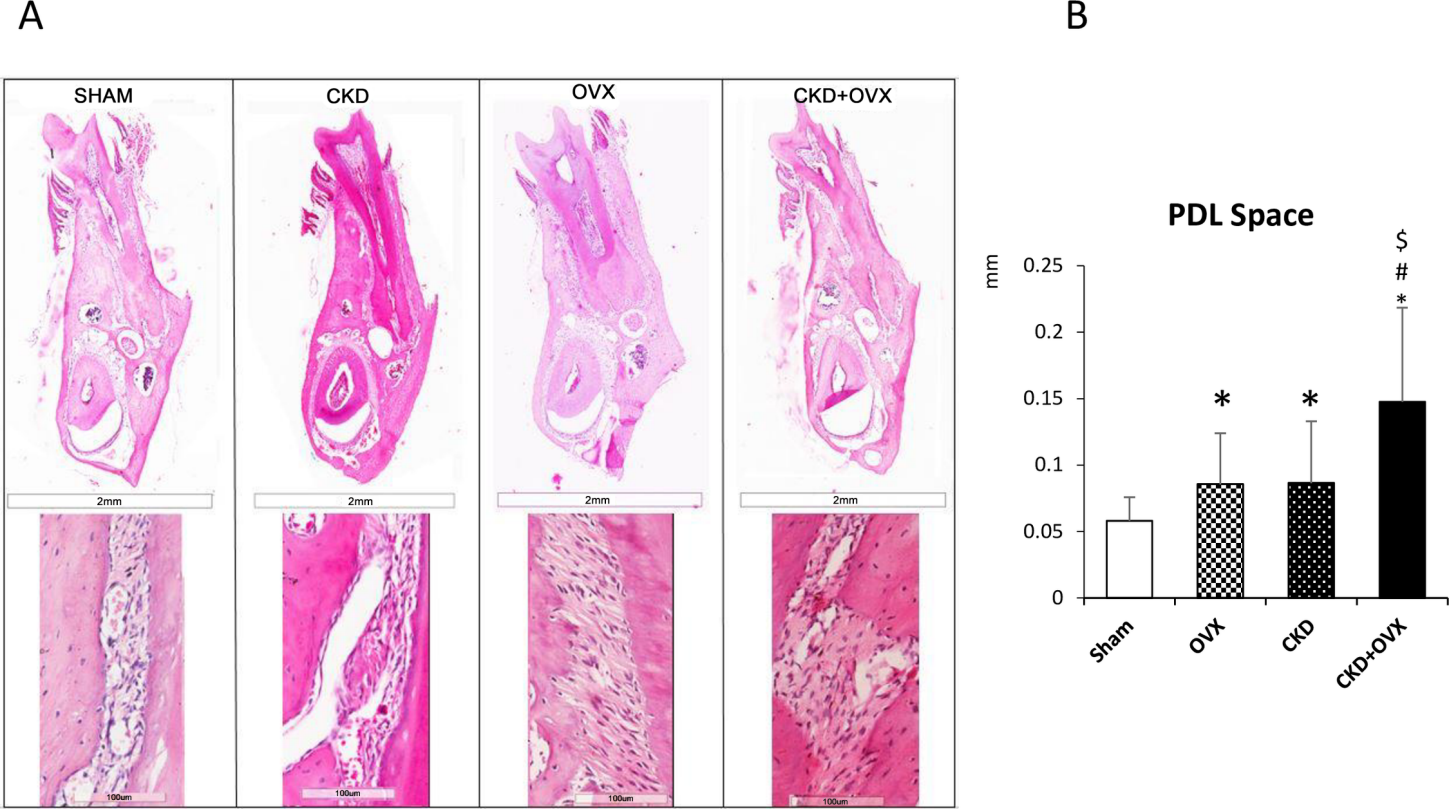
Histological staining of cortical bone and PDL space of the mesial root. (H&E staining) (A) Buccal lingual cross sections of the mesial root of the mandibular first molar. The second row consists of magnified images (400X) of cortical bone and the PDL. (B) PDL space of the mesial root. *: p<0.05 vs Sham; #: p<0.05 vs OVX; $: p<0.05 vs CKD.

One representative section from each group is shown in Figs 4 and 5. A normal trabecular bone pattern and arrangement with a few bone marrow spaces were observed in the mandibles from animals in the Sham group (Fig 4). Moreover, the Sham group showed well-organized cortical lamellar bone (Fig 5A). The PDL space was significantly wider in the other three groups than in the Sham group (Fig 5B). Furthermore, the PDL space was significantly wider in the CKD+OVX group than in the CKD group.

## Discussion

In the present study, we established a mouse model of CKD and estrogen deficiency to determine the effects of estrogen deficiency on the mandibular bone of CKD mice. Serum data showed that estrogen deficiency led to a further increase in serum BUN in the CKD mice, suggesting that OVX aggravated kidney failure. Estrogen deficiency decreased the BV/TV ratio and cortical thickness of the mandibular bone in both the OVX and CKD+OVX groups. Moreover, these declines were more severe in the CKD+OVX group than in the CKD group, indicating that estrogen deficiency led to further bone loss. Histological evaluation confirmed the increased PDL space in the CKD, OVX, and CKD+OVX groups, and the CKD+OVX group showed the greatest increase among these three groups.

We did not observe significant differences in serum FGF23 and PTH levels between the CKD+OVX and CKD groups in this study. Although serum FGF23 and PTH levels elevated as CKD progresses, they do not increase linearly with eGFR [17]. Other groups have studied the effect of OVX on serum FGF23 and PTH levels using a rat model, and in accordance with our results, no significant changes were observed [33, 40]. Moreover, serum FGF23 and PTH levels were measured at eight weeks after treatment in our study and in previous ones. As eGFR varies with time, different serum chemistry results might be obtained at other time points. Although FGF23 and PTH have direct effects on bone remodeling, the data suggest that they are not the key factors by which estrogen affects bone in CKD mice. More studies are needed to fully elucidate the mechanisms by which estrogen regulates serum FGF23 and PTH levels and affects bone in CKD mice.

CKD impairs bone metabolism and leads to CKD-MBD by reducing kidney function and disturbing the normal physiological mechanisms that regulate the blood levels of vitamin D, phosphate, calcium, PTH, and FGF23 [4–6]. The mandibular bone is involved in these dramatic changes. Using a CKD mouse model, Lee *et al*. [7] observed a significant reduction in cortical bone thickness and increases in both trabecular thickness and trabecular BV/TV. Allen *et al*. [8] also observed a significantly lower BV/TV ratio in the mandibular bone in CKD rats and reported that zoledronate and calcium administration could restore the BV/TV ratio. In the present study, micro-CT analyses showed a significant reduction in the cortical thickness of the mandible in the CKD group compared with the Sham group. However, we did not observe a significant decrease in BV/TV in the CKD group compared with the Sham group. This result is in accordance with the findings of Schober *et al*. [41], who studied bone loss in dialysis patients and observed significant cortical bone loss. CKD might affect mandibular bone and lead to significant cortical loss without considerable changes in trabecular bone. Thus, we only observed a slight decrease in BV/TV. Moreover, periodontitis is more severe in CKD patients compared with the general healthy population. Zhao *et al*. [12] observed a worse community periodontal index (CPI), more severe clinical attachment loss (AL), and greater periodontal bone loss (BL) in hemodialysis patients in China. In this study, we observed a wider PDL space in the CKD and CKD+OVX groups, and the latter group showed a more dramatic change.

Because estrogen is a critical regulator of bone remodeling, a deficiency could impair mandibular bone metabolism. Yang *et al*. [23] measured the mandibular cortical thickness of OVX rats both manually and using computer image analysis and observed a significant reduction in cortical thickness. Using micro-CT to investigate the mandible of OVX rats, they found that estrogen deficiency significantly decreased the BV/TV ratio and trabecular thickness and increased trabecular separation and the structure model index. Furthermore, estrogen deficiency affects bone healing and bone density around titanium implants [24–26]. Tateishi *et al*. [25] investigated whether estrogen deficiency interrupts bone healing around titanium implants and found a lower BIC, BA (bone area), and BD (bone density) in cancellous bone around the implants in the OVX group. Giro *et al*. [26] evaluated the influence of estrogen deficiency on bone density around implants and found that OVX led to a lower BD, which could be effectively treated with estrogen replacement therapy. Our previous study demonstrated that estrogen deficiency impairs the osseointegration of titanium implants, which is accompanied by decreases in the BIC ratio, BV/TV ratio, and biomechanical push-in resistance [24]. We also observed significantly lower BV/TV and cortical thickness in the OVX group compared with the Sham group in our current study.

Estrogen also plays a protective role in CKD patients [27–30]; thus, loss of this factor definitely accelerates the progression of CKD. Many studies have demonstrated that estrogen deficiency leads to further bone loss in CKD patients. Barreto *et al*. [32] reported that amenorrheic female patients on hemodialysis presented with a lower BV/TV ratio than menstruating patients. Weisinger *et al*. [31] reported a significantly lower trabecular BMD in the lumbar spine of amenorrheic women compared with regularly menstruating dialysis patients. In this study, we found that serum BUN levels were higher in the CKD+OVX group than in the CKD group, indicating that estrogen deficiency aggravated CKD. The BV/TV ratio and cortical thickness were significantly lower in the CKD+OVX group than in the CKD group, demonstrating that estrogen deficiency led to further bone loss.

## Conclusion

In this study, we developed a CKD and OVX mouse model to investigate the effects of estrogen deficiency on the mandibular bone of CKD mice. To the best of our knowledge, this is the first study of mandibular bone in the context of CKD and estrogen deficiency. We found that estrogen deficiency in CKD mice significantly decreased the BV/TV ratio and cortical thickness, indicating that estrogen deficiency leads to further mandibular bone loss.
